# Navigating cultural transitions during resettlement: the case of unaccompanied refugee minors

**DOI:** 10.3389/fpsyg.2023.1080072

**Published:** 2023-05-09

**Authors:** Elin Sofia Andersson, Carolina Øverlien

**Affiliations:** ^1^Norwegian Centre for Violence and Traumatic Stress Studies (NKVTS), Oslo, Norway; ^2^Department of Social Work, Stockholm University, Stockholm, Sweden

**Keywords:** unaccompanied refugee minors, acculturation, integration, cross-cultural transition, asylum-seeking children and adolescents, post-migration factors, youth development

## Abstract

**Introduction:**

Refugees face the process of cross-cultural transitions upon arrival in their host country. This process is commonly referred to as acculturation and can be particularly challenging for asylum-seeking children and adolescent unaccompanied by a caregiver. To more effectively facilitate unaccompanied refugee minors (URMs) resettlement, this study sought to obtain an enhanced understanding of the acculturation processes of these youth’.

**Methods:**

Thus, interviews with 48 URMs, all of whom arrived before the age of 16 years, were analyzed in two steps. First, how the youth described their host country’s society and culture, followed by how they navigated within this societal and cultural landscape during resettlement.

**Results:**

The youth described how they navigated the Norwegian cultural and societal landscape by gaining cultural competence, adapting and finding ways to contribute, which made it easier for the youth to gain access to the society, to succeed as well as enhance their sense of agency. However, the youths also reported having to navigate between the expectations of their original and host country cultures, struggling with finding a balance between the two cultures.

**Discussion:**

The youth’ acculturation processes seemed to be the result of both their own needs, wishes and behavior as well as specific features in their host country culture, which supports the notion that acculturation processes to some degree are context- and culture-dependent. Knowledge regarding the cultural and societal framework that these youth face and how they navigate within it during resettlement is critical for identifying possible cross-cultural challenges and promoting positive developmental tracks. To understand more about acculturation and integration processes, future research should include specific cultural and societal features as well as immigrants’ own perspectives and experiences during resettlement.

## Introduction

As of the end of 2021, approximately 36.5 million children worldwide had been displaced as a consequence of conflict and violence (UNICEF, 2022). Due to the current crisis in Ukraine, the number of unaccompanied refugee minors (URMs) is likely to increase substantially worldwide. Children and youth traveling alone are often considered particularly vulnerable refugees, both during flight and after arrival in their country of resettlement. For URMs, cross-cultural transitions can be extra challenging and, in some studies, they are associated with mental health problems (Jore et al., 2020; EL-Awad et al., 2021). Due to the lack of parental protection they are at a higher risk of experiencing stressful life events without the support from a caregiver to deal with these strains (Bean et al., 2007; Derluyn et al., 2010; Jensen et al., 2019; Pfeiffer et al., 2022). Studies report that URMs score significantly higher on internalizing problems, traumatic stress reactions and stressful life as well as having a higher risk for developing mental health disorders compared to both accompanied minors and native youth (Bean et al., 2007; Norredam et al., 2018; Müller et al., 2019). Furthermore, studies indicate that many URMs struggles with anxiety, depression and/or post-traumatic stress disorder even long after they are resettled (Vervliet et al., 2014; Jensen et al., 2019). Upon arrival, URMs face a double transition problem. They must re-establish their lives in an unknown culture as well as transition from adolescence to adulthood. As they are apart from their family during a crucial developmental period, URMs also need to establish new close relationships outside of their family (Andersson et al., 2021).

To the best of our knowledge, few studies on acculturation processes among URMs have included specific cultural characteristics and participants’ own meaning making. Therefore, in this study, we explored these questions by examining how URMs who arrive before the age of 16 years re-establish their lives and start to function in Norway, a culture different from the one they were born in and socialized into. Our investigation was in line with the idea that immigrants’ perceptions of their host country culture and society will affect their acculturation process as well as with the assumption that individual acculturation is a process executed by an agentic individual. Thus, we examined the following two research questions:

1. *How do the youth understand the Norwegian society and culture?*

2. *How do they navigate within this cultural and societal landscape during their first 5 years in Norway?*

### Acculturation

Some of the challenges for URMs upon arrival are attributed to the process of resettling in an unknown society and culture. This cross-cultural transition process, commonly called acculturation, is described as positive in terms of possibilities and safety, but also distressing and challenging. Clashes of cultural worldviews, culture shedding, ethnic identity confusion, religious differences, and discrimination have been highlighted as demands that refugees may face during their early resettlement years (Ryan et al., 2008; Andreouli, 2013).

According to Chirkov (2009) is individual acculturation a process executed by an agentic individual after entering a different cultural community. How people face this process of confluence among heritage-cultural and receiving-cultural practices, values, and identifications is commonly called their choice of acculturation strategy. However, the resulting acculturation strategy is not always chosen voluntarily. For some people it is rather a result of lack of other possibilities. According to the well-known two-dimensional model by Berry (1997), there are primarily four strategies for acculturation: assimilation, segregation, marginalization, and integration. Assimilation is where immigrants adapt to the majority culture at the expense of their original cultural identity. Segregation is where immigrants hold on to their original culture and do not interact with the majority culture, either by choice or not being given the possibility. Marginalization is where little possibility or interest exists in engaging in either the original or host country’s culture. Integration, seeking both cultural maintenance and involvement with the larger society, is the acculturation strategy associated with the best psychological outcomes (Berry et al., 2006).

Acculturation research has been criticized for mainly studying acculturation as a series of stable and mutually exclusive outcomes and not a dynamic process. Another criticism is its lack of inclusion of the diversity of migrant experiences, resulting in decontextualized and acultural accounts of acculturation (Ward, 2008; Bhatia and Ram, 2009; Andreouli, 2013). No culture is a single entity. People within the same society each create their own “personal culture” depending on how they understand their experiences and surroundings (Gamsakhurdia, 2019). Accordingly, to obtain more insights into acculturation processes, it is essential to explore how specific cultural characteristics, immigrants’ own understanding, and their positioning in their host country’s culture affect the process (Chirkov, 2009; Andreouli, 2013; Gamsakhurdia, 2018).

### Adaptation during resettlement

Several studies have explored cultural differences between original and host country cultures. The most commonly positive aspects refugees, also URMs, highlights in resettlement cultures are safety, democracy, and work opportunities (Andreouli, 2013; Woodgate and Busolo, 2021). For example, Sudanese URMs in the United States considered grasping the opportunities for education and work to be important (Luster et al., 2010).

Furthermore, many immigrants and refugees report that acquiring language and cultural competence is necessary to integrate into and participate in society. Doing so creates a better future for them and their families, which is possible through the facilitation of economic opportunities and work prospects (Luster et al., 2010; Andreouli, 2013; Fedi et al., 2019; Woodgate and Busolo, 2021). However, several studies have reported that immigrants experience the process of adaptation to be unbalanced. Immigrants have described that they alone were expected to adapt, and that their own willingness and effort were the keys to meeting the demands in the receiving society (Fedi et al., 2019; Woodgate and Busolo, 2021). Furthermore, some studies have reported that being different leads to exclusion. To be a part of a society, some immigrants adjust by copying the majority’s behavior while repressing the need to hold on to their original cultural identity (Fedi et al., 2019; Brook and Ottemöller, 2020).

Moreover, studies on URMs have demonstrated how they immigrants work hard to be involved in their host countries by creating supportive networks and developing cultural competence. Increased host-cultural competence in combination with support from one’s family abroad and the possibility to maintain one’s culture of origin are associated with fewer mental health problems and post-migration stress among these youth (Oppedal and Idsoe, 2015; EL-Awad et al., 2021). By contrast, acculturation-specific hassles, such as discrimination, feeling unsafe, and uncertainty about the future, are associated with mental health problems among URMs (Keles et al., 2016; Jensen et al., 2019). However, increased cultural competence seems to reduce discrimination levels against URMs (Oppedal and Idsoe, 2015; Jore et al., 2020).

### Pull and push between opposing cultural demands

In spite of many positive consequences of adapting to their host country cultural, many immigrants reports challenges during their cross cultural transition. For example, several studies report immigrants experiencing that their position in relation to both their original and host community are questioned during resettlement, and some are left in a position somewhere in between (Bhatia and Ram, 2001; Märtsin and Mahmoud, 2012). Subsequently, many experience ethnic identity confusion and a need to renegotiate their social representations and identities within these two positions, including a re-evaluation of cultural norms and assumptions about one’s belief systems and habits (Bhatia and Ram, 2001; Andreouli, 2013; Belford, 2017).

In particular, immigrant youth have reported tensions emerging from conflicting and opposing cultural demands and expectations between their original and host country’s ways of living (Brook and Ottemöller, 2020; Woodgate and Busolo, 2021). For example, Woodgate and Busolo (2021) reported that immigrant youth have a stronger desire than their parents to adapt to Canadian culture. Subsequently, they struggle to find a balance between these two cultural practices and experience a sense of pull and push in regard to wanting to please both their families and Canadians. This results in them not knowing whether they should keep their culture of origin, adopt the Canadian culture, or develop their own.

However, several studies have reported that immigrants face identity challenges with agency and creativity, change between positions depending on arenas or periods of their lives, create new identity positions, and/or resist being categorized. It seems as though youth are particularly likely to develop their identity by combining their ethnic differences, social networks, bilingualism, and transnationalism in several ways (Andreouli, 2013; Fedi et al., 2019).

## Methods

### Recruitment and participants

The data in this article came from a longitudinal study of URMs where all youth arrived in Norway before the age of 16 years (mean age 13.8 years). They were upon arrival placed in care centers for the youngest URMs provided by the State Child Protection Service. The participants were recruited from five of six of these care centers while waiting to be settled or returned [see Jensen et al. (2015) and Jensen et al. (2019) for a more detailed description of the original study]. Upon being granted asylum, the participants were moved into different municipalities. Here, they were often placed together with four to six other URMs in apartment units attended to by social workers. Some youth were placed in foster homes.

Qualitative interviews were conducted at two time points, namely approximately 2.5 and 5 years after arrival. Of the 95 participants in the original study, 48 youth were found and gave consent to being interviewed at both time points. The mean age of the participants at these two time points was 16.5 and 20.0 years, respectively. Furthermore, 40 (83.3%) were boys, reflecting a typical gender imbalance among URMs. The youth came from Afghanistan, Eritrea, Somalia, Sri Lanka, Ethiopia, Uzbekistan, The Democratic Republic of the Congo, Western Sahara, Chechnya, and Iraq. The majority of the participants came from Afghanistan (47.9%). All 48 youth who participated at these two time points were included in this study.

### Interviews

Open-ended semi-structured interviews were conducted in person, mainly in the participants’ homes. The interviews at the first time point concerned topics related to the interviewees’ childhood, flight, present situation, and hopes for the future. The same topics were covered during the second interview but with a stronger focus on the interviewees’ experiences of living in Norway and their present situation, friendships, aspirations, social support, well-being, agency, daily stressors, and identity. All 48 interviews were audio-recorded and transcribed verbatim. All of the youth were offered a translator, but all chose to conduct the interviews in Norwegian.

### Thematic analysis

To investigate the two research questions two different analytical steps were taken. First the interviews were analyzed according to thematic analysis and next using interpretive phenomenological analysis. To explore how the youth themselves understood and described the culture and society of Norway, interview transcripts with all 48 youth from both time points were analyzed using thematic analysis. Thematic analysis is a reflexive method that seeks to develop patterns (themes and categories) across cases. It is suitable for research on understudied topics and populations (Braun and Clarke, 2006; Braun and Clarke, 2021). To the best of our knowledge, few studies have included specific cultural characteristics when studying acculturation processes amongst refugee youth. Furthermore, although some literature has described the Nordic culture, few studies have explored how immigrants experience this culture and society. Subsequently, the thematic analysis helped us to capture the range of themes that the participants raised without limiting them to a particular theoretical perspective.

Both authors separately read and re-read all interviews and assigned codes to the transcripts. A search for patterns among the codes resulted in initial themes. These were refined through discussion, resulting in themes that presented similar topics, each of which contained several subthemes. The interviews from both time points were analyzed separately, making it possible to distinguish any potential differences due to the time in Norway. Furthermore, the numbers of youth reporting each theme and subtheme were also noted.

### Interpretative phenomenological analysis

The results of the first analysis provided us with an understanding of the societal and cultural landscape that the youth had to relate to during resettlement, as seen from their own perspectives. Considering that individual acculturation is a process executed by an agentic individual (Chirkov, 2009), our second research aim was to understand more about how the youth navigated within this landscape. Navigation, as understood by Ungar (2008, 2011), implies personal agency and motivation, as well as movement toward psychological, social, cultural, and physical resources that are required and are made available and accessible by those in power to those who are disadvantaged. The transcripts of the interviews with all 48 youth at the second time point were analyzed according to the traditional steps in interpretative phenomenological analysis (IPA) (Smith et al., 2009). At this second time point, 5 years after arrival, the youth had the possibility to look back at their time in Norway and reflect upon the process. IPA is a qualitative analysis method that examines how people make sense of major life experiences by exploring them. The researcher attempts to make sense of how the participants have made sense of their experiences, which is called the double hermeneutic (Smith et al., 2009). The second analysis aimed to grasp the participants’ own understanding and meaning making of how they navigated during resettlement. In line with this, the use of the double hermeneutic and interpretation according to IPA were considered useful for reaching, hearing, and understanding the experiences of participants, but without directly asking them about navigation among their host country’s features.

The first author took the lead in this analysis. Each transcript was read and re-read while descriptive and interpretative notes were made. Each reading focused on exploring and identifying how the youth navigated among the societal and cultural features identified in the first analysis. In this regard, the results of the thematic analysis were used as a framework of the particular cultural society that the youth had to relate to during resettlement. The interviews were first analyzed vertically one at a time, followed by horizontally across interviews. The notes taken were developed into emerging themes for each participant before patterns and connections were searched for across all participants. The final themes were named to reflect their conceptual nature and mirror the youth’ words and thoughts as well as the researchers’ interpretations. The first author analyzed all of the interviews. The second author read all of the interviews and analyzed randomly selected ones to reduce interpretative bias in the analysis (Hill et al., 2005). The analyses were then compared, any disagreements were discussed, and a consensus was reached.

### Ethics statement

This study was approved by the Regional Committees for Medical and Health Research Ethics and has therefore been performed in accordance with the ethical standards laid down in the 1964 Declaration of Helsinki and its later amendments. All youth gave their informed written consent prior to their inclusion in the study. For those under the age of 16, written informed consent was also provided by their legal guardian. All audio recordings and transcriptions were stored in a secure data storage system only accessible to the researchers. Direct quotations were translated and used as examples to illustrate the participants’ perspectives. All names were altered and details that might disclose the identity of the subjects under study have been omitted to ensure confidentiality.

## Results

### How do the youth understand the Norwegian society and culture?

**Figure 1:**
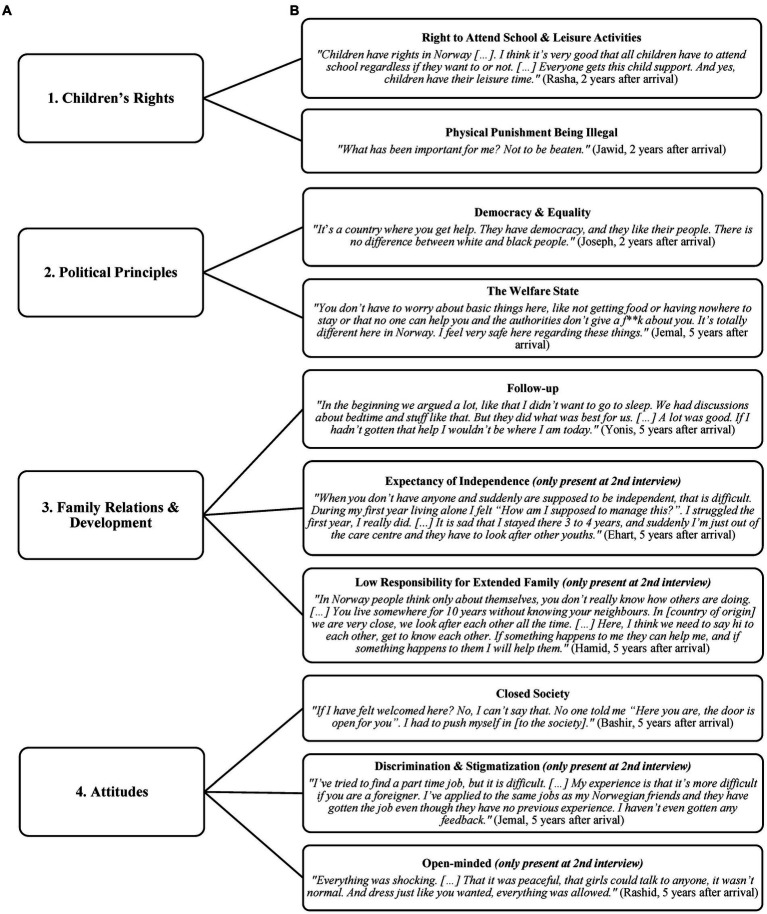
A graphical presentation of the results from the thematic analysis which explored how the youth understood the Norwegian society and culture. The four themes **(A)** are presented to the left and the subthemes **(B)** to the right along with quotations for each subtheme.

The first analysis, which explored how the youth understood the culture and society of Norway, resulted in the following four themes: *Children’s Rights, Political Principles, Family Relations & Development*, and *Attitudes*. Each theme contained several subthemes. All themes and subthemes present at the first interview were also present at the second interview, whereas some subthemes were only present at the later time point. The findings for each theme are presented in the following subsections. See Figure 1 for a graphical presentation of the themes and subthemes along with quotations for each subtheme.

#### Children’s Rights

The two subthemes of *Right to Attend School & Leisure Activities*, and *Physical Punishment Being Illegal* constituted the main theme of *Children’s Rights*. Regarding *Right to Attend School & Leisure Activities*, a majority of the youth at both time points highlighted that attending school was not only possible but also mandatory. This was often discussed in contrast to the situation in their countries of origin, where school access was highly limited. Those youth who at the second interview had finished high school talked in hindsight about how important it had been to receive an education and what opportunities it had given them. The youth also highlighted their right to participate in leisure activities, and in that regard being children. In regard to *Physical Punishment Being Illegal*, a majority of the youth described experiencing violence from caregivers and/or teachers in their country of origin. After arrival in Norway, the youth expressed that they had felt disbelief but also a great relief that no physically punishment was allowed.

#### Political Principles

The theme *Political Principles* entailed the two subthemes of *Democracy & Equality* and *The Welfare State*, both of which were present at the first and second interviews. A few youth in the first interview elaborated on what they experienced as democratic values, such as equal rights and that the state and politicians can be trusted. In the second interview, even more youth described their experiences with democratic values in Norway. Features such as individual freedom, human rights, political and religious freedom, as well as equal rights independent of gender, ethnicity, and sexual orientation were highlighted.

Included in the subtheme of *The Welfare State* were the youth’ descriptions of their basic needs, such as clothes, food, housing, and access to health care services, being provided and facilitated by the state. Receiving so many benefits seemingly out of nowhere created a sense of being valued and cared for. However, bureaucratic processes were also described as exhausting and frustrating. A few youth had experienced how crucial decisions were made without them having a say or that their wishes had not been heard or respected, such as where to live and who to live with. Others had experienced responses to their applications, such as residence permit, financial help or a place to stay, taking a very long time, leaving them to agonize over time.

#### Family Relations and Development

The following three subthemes were included in the theme *Family Relations and Development*: *Follow-up*, *Expectancy of Independency*, and *Low Responsibility for Extended Family*. The subtheme of *Follow-up* was present in the interviews at both time points, although more youth talked about it during their second interview. Social workers and foster parents were described as providing comfort, help, support, and guidance toward managing daily life, but also as setting strict rules and limits. Setting limits was often frustrating for those youth who had lived very independent lives. A majority of the youth had to take care of themselves for a long time during their escape, and/or they were given greater responsibility in their countries of origin, such as working to help provide for their families or looking after younger siblings.

Descriptions related to the subtheme of *Expectancy of Independency* were primarily present in the second interviews. Five years after arrival, a majority of the youth described having experienced a strong expectancy of being independent when they reached their legal age. The youth described how they went from receiving much help to almost none, and also how difficult it was to suddenly manage everything by themselves. However, a few youth talked with pride about how they had learned everything they needed to be independent and able to fend for themselves.

The subtheme of *Low Responsibility for Extended Family* was only present in the youth’ stories in the second interviews. This subtheme entailed the youth’ experiences with people in Norway having scant responsibility for other people in their local community, such as family, extended family, and neighbors. These youth missed and longed for a sense of belonging to a greater network with those closest around them, where people looked after and helped each other if required.

#### Attitudes

The following three subthemes were included in the theme of *Attitudes*: *Closed Society*, *Discrimination & Stigmatization*, and *Open-mindedness*. In regard to the subtheme of *Closed Society*, youth at both time points reported how they experienced Norwegians as closed in regard to being hesitant or not interested in interacting and establishing new relations. This made it difficult for the youth to make friends and create a network.

In regard to the next subtheme, it was primarily during the second interviews that the youth described both indirect and direct experiences related to being discriminated against and stigmatized. A few youth reported receiving racist verbal abuse in public or being treated differently by public officials such as health workers and the police. A majority of the youth described that they had experienced a general skepticism toward immigrants, resulting in them experiencing difficulties in obtaining a job, for example.

The subtheme of *Open-mindedness* entailed the youth’ experiences with Norwegians being more liberal, primarily in regard to relationships, showing affection in public, gender roles, and codes of dress and behavior. In particular, women were described as being freer to dress and behave as they wanted, but men also experienced more freedom in regard to choosing their own clothes and hairstyles.

### How did the youth navigate in this landscape during their first 5 years in their host country?

The participants in this study were young adolescents when they came to Norway. It was this particular cultural society, as presented in the first analysis, that they had to relate to during resettlement and their critical period of transition between childhood and adulthood. In line with the idea that individual acculturation is a process executed by an agentic individual after meeting and entering a new and different cultural community, we asked the question: *How did the youth navigate in this landscape during their first 5 years in their host country?* The analysis resulted in the following three themes that were all keys to a fruitful acculturation process during these first years: *Succeeding by Understanding and Adapting*, *The Importance of Contributing*, and *Balancing Between Expectations*. In the following subsections are the findings for each theme presented along with selected quotes.

#### Succeeding by Understanding and Adapting

A majority of the youth expressed being overwhelmed by the huge differences between their original and host country culture upon arrival, and many struggled with this cross-cultural transition. Despite this, all of the youth in this study were set on succeeding, which ultimately meant being a part of the society in terms of having friends and being self-supportive through work. These ambitions were a significant driving force when navigating during resettlement. A majority of the youth, such as Akmal, soon realized that understanding and adapting to the culture was crucial for achieving their goals:

“You need to understand the society and walk that path. If you behave just like in [country of origin], that doesn’t work in Norway. It’s very important to understand the Norwegian culture.” (Akmal, 5 years after arrival)

In this study, the youth described several features of their host country’s society that aided them in achieving their goals. For example, being provided with basic needs such as housing, food and clothing as well as free schooling meant that they could focus on their education instead of having to work (subthemes *Welfare State* and *Right to Attend School & Leisure Activities*). Receiving an education was critical for all of the youth, who viewed it as the primary gateway to their goal of succeeding through work. However, attending school was not always that easy. Upon arrival, none of the youth knew the local language and a majority had scant schooling, making attending the same classes as their peers challenging. Furthermore, a majority of the youth had experienced violence from teachers prior to their arrival. A few youth said that initially upon arrival they did not want to attend school as they were scared of being hit. Reassurance that they would not receive physical punishment helped them to acquire a sense of safety in school (subtheme *Physical Punishment Being Illegal*). Furthermore, hard work was crucial for achieving their goals, as was their willingness to use the opportunities and help provided by those around them (subtheme *Follow-up*).

On their way to becoming adults in Norway, the youth leaned on the systems of care and legal rights for children. However, they described some features of their host country’s society as making the achievement of their goals more difficult. A majority of the youth reported that it was especially crucial to understand and navigate prejudices, discrimination, and Norwegians being careful in establishing new relations (subthemes *Closed Society* and *Discrimination & Stigmatization*). For example, understanding this hesitancy as a cultural feature made it easier to not take it personally. It was also helpful in realizing how to navigate this, as Asiba described:

“I thought in the beginning that people were really ignorant. Because where I’m from, if you are new, people will come to you and talk to you so you won’t feel alone. […]. But here, you need to do it yourself, no one will come to you.” (Asiba, 5 years after arrival)

Like Asiba, other youth realized that they themselves needed to engage and take the initiative to get in contact with people and make new friends. However, part of their navigation also involved realizing the right balance between initiating contact and appearing too pushy, as was exemplified by Akmal as follows:

“I’ve changed a lot. I used to be very open, just talking about anything. But you can’t do that in Norway. For example, once when I was out with friends I said hi to someone. He got really angry: “Do you know me?”. I didn’t mean anything bad, I just like to talk to people. […] But I learned and I’ve changed, I’ve become Norwegian. I’ve become integrated and I understand the society. I adapt, but the old personality is still there inside me.” (Akmal, 5 years after arrival)

As portrayed by Asiba and Akmal, these youth needed to tread carefully to fit in, taking initiative but not being overly assertive. Finding the right balance and adapting their behavior accordingly made it easier to make friends. Making local friends was nevertheless still described as challenging.

In addition, the youth reported being treated negatively and differently because they were foreigners (subtheme *Discrimination & Stigmatization*). The most common events reported were being met with a general skepticism and prejudices because of their ethnicity, having more difficulties gaining a job compared with their local peers, and receiving stigmatizing comments. A few youth even reported having racist abuse yelled at them in public. These experiences made it challenging for them to make friends as well as to find a job. A majority of the youth discussed how they navigated these situations by trying not to care, being like those around them to fit in better, and working very hard and not giving up until they had reached their goals. Faraz described how getting a job had been very difficult for him compared with his local friends, but how he had continued trying until he achieved his goal:

“I think that when I want something, I’ll never give up. […] I remember the first time I applied for a job. It was really difficult, and I had to continue trying. Every day after school, I asked to talk to the manager if there were an opening. And when you show a lot of interest, you will eventually succeed.” (Faraz, 5 years after arrival)

After gaining cultural competence and adapting to the majority’s behavior, a majority of the youth reported experiencing greater possibilities to make friends and find a job. However, adjusting their ways of being was for some youth inevitably followed by having to repress their own cultural identity more than they actually wished to.

#### The Importance of Contributing

A wish to help other people and contribute to society was evident in a majority of the youth’ stories. Throughout their flight and resettlement, all youth had experienced being in a vulnerable position. Upon arrival in Norway, the youth were provided with basic needs such as food and housing, free schooling, as well as help and support from social workers (subthemes *Right to Attend School & Leisure Activities*, *Follow-up*, and *Welfare State*). Receiving all of these goods for free, without people knowing them or wanting something back, was surprising and strange, and they could almost not believe it. Furthermore, with time in Norway, the youth experienced the government as trustworthy and aiming to help the country’s inhabitants (subtheme *Democracy & Equality*). In the analysis, it became evident how much the youth appreciated and were grateful for these things. In their efforts to be part of the society, they worked hard to find ways to give back and help others. As exemplified by Ahmed, one way of doing this was to focus on working and paying taxes so that others would have the same benefits as he had been given:

“My goal is to help those that helped me. I’m thinking, they paid taxes so that I could go to school, see the doctor and everything like that. So now, those who paid taxes will get help from me.” (Ahmed, 5 years after arrival)

Other youth engaged in volunteer work. Thabo was only 19 years old when he applied to the local firefighter team, and to date he remains the youngest member to be recruited:

“I like contributing to society. I volunteer to coach children soccer in [town]. Now, I also volunteer at the local fire station since they needed people. I was thinking, I live here, I play soccer here, my friends are here, so why not?” (Thabo, 5 years after arrival)

By paying taxes and participating in voluntary work, the youth experienced that they were appreciated and an important part of society. Navigating toward giving back to society and helping others turned out to be crucial keys into the Norwegian society. Moreover, being able to also contribute was critical for the youth’ own sense of agency.

#### Balancing Between Expectations

The third theme that evolved during the analysis of how the youth navigated in the described landscape during resettlement was how to balance between different expectations, both within and between their original and host country cultures. As presented in the first theme, gaining cultural competence and adapting their ways of being were not only crucial to succeed but also important keys for gaining access to the society. However, when adapting, the youth risked negative reactions from people from their culture of origin. Maher described how he had experienced this balancing struggle:

“The way of living in [country of origin] and in Norway, which should I choose? How should I balance it? […] If you do something typically Norwegian, people from [country of origin] will say “Oh, so he has become Norwegian?” At the same time, Norwegians wants us to adapt to Norway. If you do like Norwegians wants, people in [country of origin] will be dissatisfied. Now I’m thinking, I don’t belong anywhere. […] I’ve become in between.” (Maher, 5 years after arrival)

As exemplified in Maher’s story, a majority of the youth needed to navigate the different – and often conflicting – expectations from their original and host country cultures, but it was often impossible to satisfy both. On the one hand, adaptations seemed to be necessary to function in their everyday lives in Norway. On the other hand, adjusting their ways of being inevitably led to moving away from their culture of origin and risking being rejected. As a result, the youth found themselves being pulled in two different directions simultaneously and they struggled to find a balance between these two positions. At worst, this led to a feeling of not belonging anywhere. Although a majority of the youth did not report feeling totally excluded from both cultures, all of them had to reflect upon how to navigate between the expectations of their society and culture of origin and their host country’s culture and society. While some youth felt a greater belonging to their culture of origin, others felt that Norway had become their home country. Like Ehart, some resolved this dilemma of navigating between two different cultures by combining them to create their own culture:

“There are many good things in the Norwegian culture. And then I have my own culture as a role model. I pick some things and put in the Norwegian culture, mixing them for myself.” (Ehart, 5 years after arrival)

As reflected in Ehart’s story, a critical part of this process all youth had to go through was to reflect upon which behaviors, values, and norms to keep; which to let go of; and which to embrace; and ultimately how to combine them. Some societal and cultural features in Norway, such as school being accessible for all, no physical punishment, and everyone having equal rights (subthemes *Right to Attend School & Leisure Activities*, *Physical Punishment Being Illegal*, *Democracy & Equality*, and *The Welfare State*) were embraced by a majority of the youth. However, in contrast to the subtheme *Low Responsibility for Extended Family*, most youth worked hard to keep and create a sense of mutual responsibility toward those around them. These efforts could entail maintaining close contact with and feeling responsibility toward their family and/or working to establish a sense of mutual responsibility in their local area with friends and neighbors.

Moreover, a majority of the youth reported being exposed to violence at home and/or in school before arrival (Jensen et al., 2019). Arriving in Norway, they learned that hitting children and youth is harmful and thus illegal, which brought a sense of relief (subtheme *Physical Punishment Being Illegal*). However, this knowledge also created a discrepancy for those youth who described receiving love from their parents despite being physically punished. They experienced that it was done with the best of intentions, specifically to teach them the difference between right and wrong. In light of this knowledge, these youth needed to make sense of their previous experiences. In Samir’s meaning-making process, he compared the conditions for raising children in his culture of origin with those in his host country’s culture:

“In Norway, you grow up the right way, learning what to do and not without being beaten. But it wasn’t like that where I am from. […] They don’t have the education to teach children the right way, so they use the knowledge they have, and that is physical punishment. […] I try to explain that they shouldn’t do that […] that there is other ways than beating […] I try to show the right way.” (Samir, 5 years after arrival)

While acknowledging that what his parents did was wrong, Samir also acknowledged that they had the best of intentions and did the best they could based on their knowledge at the time. Furthermore, he described how he attempts to pass his new knowledge along to his parents in an effort to ensure that his siblings do not have to experience what he did.

Another issue during resettlement that the youth highlighted was navigating between close help and support for children and adolescents and the expectation of managing by oneself after becoming an adult (subthemes *Follow-up* and *Expectancy of Independency*). As the youth arrived in Norway in their early adolescent years, the cultural features making sure they had close help and support were often described as vital for being able to re-establish and succeed in their host country. However, the type and degree of help and support that they received, and especially all the rules and limits, were to some extent also experienced as frustrating and making no sense. Thus, navigating such situations often ended in arguments and fights, as depicted by Arman as follows:

“I argued a lot with them over everything they said no to. I thought they were mean. […] I thought, I’ve managed to get through so many countries on my own, and today he says I can’t be out until 11 pm. Then I thought he was just being mean.” (Arman, 5 years after arrival)

Instead of feeling supported and cared for, some youths felt mistrusted and mistreated, which induced a feeling of lost agency among the youths. Many struggled with balancing between both wanting and needing close follow-up to be able to move forward with their lives and at the same time experience some of it as excessive and belittling. In some ways, navigating this type of close follow-up goes hand in hand with the expectation of being independent once one becomes an adult (subtheme *Expectancy of Independency*). The transition from adolescence to adulthood was sometimes described as challenging and characterized by a sudden shift from much help and support to suddenly having to manage on one’s own. In addition, the youth experienced little help or support from their local communities (subtheme *Low Responsibility for Extended Family*), leaving them with a feeling of being on their own. Jawid described how he navigated this situation:

“I need to work hard, I can’t go to school just for fun. I need to study and move forward, because they will eventually say goodbye. And that is what I did. I did my homework, exercised to be healthy, and asked a lot about rules and the system. Learned as much as possible from them.” (Jawid, 5 years after arrival)

As exemplified by Jawid here, by using the help and support provided (subtheme *Follow-up*), the youth navigated these circumstances by working hard to prepare for this transition by gaining sufficient knowledge and acquire the skills and knowledge necessary to manage everyday life on his own.

## Discussion

With this study, our aim was to obtain an enhanced understanding of URMs’ acculturation processes during resettlement from the youth’ own perspectives. This was done by first analyzing interviews with URMs on how they understood the Norwegian society and culture and thereafter how the youth navigated within this cultural and societal landscape.

Norway is one of five countries in the Nordic region. Often highlighted as core elements in these countries are a universal welfare state, egalitarian values, a strong and liberal democracy with strong civilian, and especially children, rights (Hvinden, 2009; Doksheim, 2011; Doksheim, 2017). Several of the themes and subthemes highlighted in the youth’ descriptions of Norway were in line with research describing the Nordic countries. In that regard, they were not surprising. However, culture is not a single thing, and people within the same society can experience different cultural characteristics (Gamsakhurdia, 2019). In line with this, the themes and subthemes presented in the first analysis can be regarded as representing how these particular youth as URMs in transition from adolescence to adulthood experienced their host country culture and society during their first years of resettlement. Considering that being a part of a culture and a society is based on each individual’s understanding of that culture, these results were an important foundation for the second analysis, which focused on how the youth navigated within this particular landscape.

In the second analysis, it became evident that *how* the youth in this study navigated during resettlement in some ways was reliant upon specific features in their host country culture. For example, finding the right balance between taking initiative to contact and not being too outgoing seemed to be critical behavioral adjustments for making friends. However, this particular balance would probably not be a crucial adjustment in countries where being careful about establishing new relations is not such a strong norm. Furthermore, the youth experienced that navigating toward paying taxes and volunteer work were keys into the Norwegian society. This is in line with the long tradition in the Nordic countries of a welfare state and a high degree of trust that paying taxes will benefit the inhabitants (Doksheim, 2011; Andreasson, 2017).

Together, the two analyses in this study provided an impression of these particular youth’ acculturation processes, considering their subjective experiences regarding specific host country societal and cultural features as well as the youth’ experiences with navigating among them. According to the results, the youth’ acculturation process entailed both possibilities in the form of heightened safety, work, and education, but also challenges such as how to become a part of the society and where to belong. In light of this, this section further elaborates on the youth’ experiences regarding integration and agency during their resettlement.

### Integration and acculturation

As became evident in the second analysis, a majority of the youth experienced that seeking involvement with the Norwegian society by gaining cultural competence, adapting, and contributing made it easier to gain access to the society and succeed. The youth’ aspiration toward gaining cultural competence and engaging in activities that facilitated opportunities for work and a better future is in line with research literature. Both youth and adult immigrants, also URMs, have expressed the importance of engaging to understand and gain knowledge about their host country’s culture (Luster et al., 2010; Oppedal and Idsoe, 2015; Fedi et al., 2019; EL-Awad et al., 2021; Woodgate and Busolo, 2021). Andreouli (2013) reported that cultural competence and adjusting their behavior, among other things, increased immigrants’ chances to participate in the society, thus facilitating opportunities for work and a better future. In this study, the youth strive toward and, at least for many, succeeding in being part of the society by adjusting and contributing is contradictory to how immigrants and refugees, and especially non-Western migrants, often are portrayed in the media. Media news on immigration is often negative, focusing on immigrant and refugees physical, economic and cultural threats to host societies and how their values and norms are incongruous with their host societies (Esses et al., 2013; Eberl et al., 2018; Cengiz and Eklund Karlsson, 2021). The findings in this study seems to support the notion that non-European immigrants can integrate well into Western cultures. Although the youth repeated efforts to gain access to the Norwegian society should not be underestimated, cultural adaptation can in some ways be easier for youth, considering that their identities are not so established and that they have more educational and socialization opportunities (Pumariega and Rothe, 2010; Cheung et al., 2011). Integration, seeking both cultural maintenance and involvement with the larger society, is the official political aim in the Nordic countries when receiving migrants and refugees (Nordisk Ministerråd, 2020; IMDI, 2022; Nordic Co-operation, n.d.). This is also the acculturation strategy associated with the best psychological outcomes (Berry et al., 2006). By contrast, the youth in this study occasionally experienced little room for actual cultural maintenance due to some societal and cultural features in Norway. Furthermore, they experienced that integration according to the society of Norway is to a large extent conditional on their ability or willingness to adjust their ways of being. Other studies have reported similar experiences of adjustment expectations, with immigrants sometimes having to adjust to such an extent that it is difficult to hold on to their original identity and culture (Fedi et al., 2019; Brook and Ottemöller, 2020; Woodgate and Busolo, 2021). In this regard, the expectation of a high degree of adjustment might be present across different host countries and immigrant age groups, not only in Norway. However, having to adjust to such an extent that one must let go of one’s original culture is not in accordance with the concept of integration, which entails both cultural maintenance and involvement with the larger society. This seems rather more like being pushed into the acculturation strategy of assimilation, which includes seeking belonging to the majority culture without maintaining one’s original cultural identity (Berry et al., 2006).

In addition to the necessity to adjust and adapt, the youth had to relate to expectations of holding on to their culture of origin, as was evident in the theme *Balancing Between Expectations*. Consequently, they struggled to find a balance between expectations from their original and resettlement societies. This is in line with several studies that have described immigrant youth experiencing tension from conflicting demands from their original and host country cultures, resulting in a sense of pull and push (Ryan et al., 2008; Brook and Ottemöller, 2020; Woodgate and Busolo, 2021). Wanting to please both resulted for some of the participants in this study in not knowing whether they should keep their culture of origin, adopt that of their host country, or develop their own, which is in line with (Woodgate and Busolo, 2021). That the youth had to struggle – and sometimes choose – between these two cultures is not in accordance with the concept of integration. Furthermore, the youth experiencing these conflicting demands is concerning considering that research has indicated that host-culture competence together with maintenance of one’s culture of origin and support from family abroad are associated with fewer mental health problems and less post-migration stress (Oppedal and Idsoe, 2015; EL-Awad et al., 2021). Discussions about resettlement and integration are most often held from the host country’s perspective. However, the youth experiences with adapting, integrating and balancing between expectations highlights the importance of also including immigrants’ perspectives and experiences in such discussions. Doing so will provide valuable information regarding the possibilities and limitations in acculturation processes as well as the host country’s responsibilities.

As presented, the youth’ stories also highlighted features that made integrating more difficult. For example, they experienced getting in contact with locals as more difficult and they were exposed to prejudices and rejection due to their race and ethnicity (subthemes *Closed Society* and *Discrimination & Stigmatization*). The youth’ experiences with discrimination were only present in the second analysis. This may be related to individual challenges they faced as they grew older in combination with different social and cultural features in Norway becoming more or less salient depending on their age. For instance, in Norway, there is a strong cultural ideal that children and youth should be protected and taken care of (Hvinden, 2009, Doksheim, 2011, 2017). Providing refugee children education and safe housing, preferable in a stable home, does thus not create discussions or protests in Norway because it is seen as a child’s rights. Although being minor at the time of the first interview, most participants had by the time of the second interview, 5 years after arrival, reached legal age and were thus considered as adults. Many had finished mandatory schooling and were applying for jobs. In other words, they were in the midst of transitioning into adulthood. It was at this time in their life and while facing these new life circumstances many of them reported experiencing discrimination. Adult refugees in Norway are not as protected by the social and cultural features of children’s rights as minor refugees are. For the youth in this study, it seems like they, 5 years after arrival, were confronted with prejudices and stereotypes as other adult migrants in Norway. Although structural racism is present in Norway as in many other European countries, it seems as if minor refugees, and maybe unaccompanied in particular, have a window of opportunity when they first arrive and (still) are protected by the strong cultural ideal of children having rights of their own and needing protection. If they during this time learn important keys to become a part of the society, integration may become easier than for adult refugees. However, this does not mean that minor refugees do not face racism and discrimination. For example, the youth in this study reported already at the first interview that they experienced Norway as a closed society, and subsequently that it was difficult to get Norwegian friends. As they grow older they may understand and interpret this as a form of racism. While acculturation-specific hassles such as discrimination are associated with mental health problems among URMs is increased host country cultural competence associated with less discrimination (Keles et al., 2016; Jensen et al., 2019; Jore et al., 2020). For the youth in this study, this is an indication of the potential consequences of experiencing and navigating features in the subthemes of *Closed Society* and *Discrimination & Stigmatization*, and the importance of understanding and adapting. Furthermore, closely linked to the issue of discrimination as well as to class and gender, is the issue of race. Considering that the type of racial discrimination an immigrant experiences depends on an interaction between their different social identities, Ball et al. (2022) suggests that an intersectional approach is important to fully understand the complexity of discrimination against minority persons. With this perspective, one might capture the complexity of how racism is intertwined with other forms of discrimination such as gender, age, class, sexual orientation and origin. In future studies, an intersectional approach would be fruitful to even better capture the complexity of types of discrimination URMs experiences.

In sum, the youth reported several societal and cultural features that made integrating more difficult, but also features in Norway that made resettlement easier (e.g., features present in the subthemes *Right to Attend School & Leisure Activities*, *The Welfare State*, and *Follow-up*). It might be that some societal and cultural features in Norway facilitate integration on a structural level while some hinder it on a more interpersonal level. In light of this, it is evident that these youth’ acculturation processes occurred in the context of both their original and host country societies and cultures and the possibilities and limitations imposed by these surroundings. In this regard, the type of acculturation strategy and being integrated are not only up to the youth themselves. This supports the notion that acculturation processes are context- and culture-dependent, as has been argued by Bhatia and Ram (2009) and Ward (2008). Furthermore, the youth’ understanding of Norway changed with time. In light of this, it seems as these processes are ongoing and to some extent change with time and developmental needs, which indicates that acculturation processes are dynamic (Ward, 2008; Bhatia and Ram, 2009). However, acculturation processes also seemed to a large extent to be affected by the youth themselves, their needs, and their wishes. The youth’ agency during transition is discussed in the next section.

### Agency in transition

In line with research literature, a majority of the youth in this study reported how they upon arrival struggled with the cross-cultural transition and the huge societal and cultural differences (Ryan et al., 2008; Andreouli, 2013; Jore et al., 2020; EL-Awad et al., 2021). In spite of this, agency and creativity were evident throughout the youth’ acculturation processes when they navigated the Norwegian societal and cultural landscape. For example, they repeatedly observed, imitated and would not give up until succeeding in their efforts to gain access to the society through contributing, understanding and adapting.

Furthermore, as presented, the youth needed to balance between expectations from their original culture and host country cultures. Several studies indicate that immigrants, particularly youth, face this balancing with agency and creativity; for example, they change between positions, combine identities, or create new ones (Andreouli, 2013; Fedi et al., 2019). This is in line with the youth in this study. Although challenging, a majority faced this sense of pull and push between the two cultures with agency, hard work, and creativity, finding a way to be a part of both cultures. In accordance with Belford (2017), they re-evaluated cultural norms, belief systems, and habits, and tried to combine different parts of the cultures, making their own (Belford, 2017). Creating their own culture is in line with the study of Luster et al. (2010), in which URMs in the United States reported combining the best aspects from their Sudanese culture with the best of American culture. These processes were nonetheless described as challenging, and a feeling of never being good enough for both cultures sometimes emerged. These experiences are in line with relevant literature. Ethnic identity confusion and a sense of being in between have been described as common during acculturation, resulting in immigrants often having to renegotiate their identities (Bhatia and Ram, 2001; Ryan et al., 2008; Märtsin and Mahmoud, 2012; Andreouli, 2013). Evidently, this was also a critical theme in the acculturation processes for the majority of youth in this study.

In addition, there were situations during resettlement where the youth experienced that their sense of agency was inhibited. In Norway, the youth sensed a strong cultural ideal that children and youth should be protected and taken care of (as was evident in themes and subthemes such as *The Welfare State*, *Children’s Rights*, and *Follow-up*). In Norway, caregivers closely monitoring and implementing rules and limits during children and youths’ upbringing, such as curfew, bedtime, and restricting time spent on the Internet, is common and deemed responsible caregiving. However, the youths in this study were raised in societies with less close follow-up and had to a large extent managed themselves during their flight through many countries. In light of this, this type of follow up made no sense. Rather than feeling cared for, some felt mistrusted or mistreated and with a diminished sense of agency. The conflicts and frustrations that these situations created can thus be understood as the youth’ way of enhancing their sense of agency. On the one hand, URMs have the right to the same follow-up as their peers in Norway. On the other hand, it might be that these youth have a greater need to be understood through their previous experiences. If the type of care, support, and follow-up to some degree were adjusted to fit these particular youth and their previous experiences, this might reduce friction and conflicts as well as help the youth to feel empowered rather than like they have lost agency.

In the Nordic countries, learning autonomy and self-determination is considered necessary to develop into independent adults (Hellevik, 2005; Nordisk Ministerråd, 2020; Skivenes, 2020). This ideal was reflected in the youth’ descriptions of having to be independent when becoming an adult, which affected the youth’ sense of agency in different ways. The youth who learned necessary skills and knowledge described a sense of thriving once becoming independent. However, others experienced a sudden loss of help without being ready, thus experiencing a loss of agency. This sudden loss of support might be a result of growing up in institutional care, where the state is no longer responsible for follow-up when one becomes an adult. By contrast, most youth born and raised in Norway experience a “transition phase,” where caregivers gradually decrease their involvement and follow-up from adolescence into adulthood (Hellevik, 2005). Furthermore, youth’ experiences of being on their own might be reinforced by their experiences of low involvement with their local communities, such as neighbors and family (subtheme *Low Responsibility for Extended Family*). This is in accordance with relevant literature that has described a strong tendency of defamiliarization in the Nordic countries. Accordingly, it is the state, not one’s family, who is considered to have provider responsibility. This diminished private responsibility has been criticized for resulting in, for example, too little responsibility for those around you and family members (Hellevik, 2005; Doksheim, 2011; Doksheim, 2017). It seems that many youth in this study were used to a greater sense of belonging and responsibility toward those around them. Not having their family or childhood friends around, many expressed a longing to receive this kind of support, but also the opportunity to be there for others and help others.

The framework for this study involved the assumption that individual acculturation is a process executed by an agentic individual. Clearly, the youth showed a lot of agency during their resettlement, which affected their own acculturation process. There also seemed to be several features that facilitated or limited their sense of and possibility to influence these processes.

## Conclusion and implications

As URMs are an especially vulnerable group of refugees, enhanced knowledge about their acculturation processes might make it easier to more effectively facilitate their resettlement in their host country. By analyzing interviews with URMs, it became clear that they navigated the Norwegian cultural and societal landscape by working hard to understand and adapt, to contribute to the society, and balance between different cultural expectations. The youth’ acculturation processes seemed to be the result of both their own needs, wishes and behavior as well as specific features in their host country culture, which supports the notion that acculturation processes to some degree are context- and culture-dependent. This highlight the importance of considering specific cultural and societal features when grasping acculturation processes. Subsequently, knowledge regarding the cultural and societal framework that these youth face and how they navigate within it during resettlement is crucial for identifying possible cross-cultural challenges and promoting positive developmental tracks.

For those working closely with immigrant youth in their daily lives, such as social workers, legal guardians and foster parents, it is crucial to take these experiences seriously. At arrival, it is important to provide children and youth with information regarding their host country culture and facilitate reflections regarding differences and similarities between their original and host country cultures. Doing so might help the youth to deal with culture shock and provide them with valuable information about how to navigate during resettlement. For example, information that Norwegians tend to be hesitant in interacting and establishing new relations is a cultural trait may be important for the youth to know when trying to establish new relationships. Furthermore, to make it easier for the youth to make friends and create a sense of community, it is important that social workers facilitate participation in arenas such as school and leisure activities.

To increase the youth’ chances for employment when becoming an adult, it is essential they receive help to do well in school. With time, it is important to facilitate for more work-related measures, such as helping them to get a drivers license or a part-time job. On a societal level, the Norwegian society may mitigate work-related discrimination by increase research on racism and discrimination on the grounds of ethnicity and religion. More knowledge on the complexity of discrimination will make it easier to develop specific interventions. Furthermore, campaigns to combat hate and harassment in the workplace, strengthening the equality and anti-discrimination commissioner as well as campaigns on how to file complaints about discrimination based on ethnicity and religion may be effective. Considering that discussions about resettlement, integration and discrimination are most often held from the host country perspective, this study points to the importance of also including immigrants’ perspectives and experiences in such discussions.

The youth in this study reported struggling with, often conflicting, expectations from their original and host country societies. In light of this, it is crucial that adults close to these youth help them to elaborate on how they can relate to and be part of both cultures. For example, older URMs or other migrants, who themselves have gone through a similar process, can provide the youth with examples of how they have dealt with this. This is especially important considering that the possibility to engage in both original and host country culture is associated with fewer mental health problems and less post-migration stress. However, just as important is that the youths are encourage to reflect upon how *they* want to relate to these different cultures, and what they want to embrace and possibly let go of.

## Data availability statement

The datasets presented in this article are not readily available because of the sensitivity of the material and the impossibility of anonymization. Requests to access the datasets should be directed to e.s.andersson@nkvts.no.

## Ethics statement

The participants gave their informed written consent prior to their inclusion in the study. For those under the age of 16, written informed consent was also provided by their legal guardian.

## Author contributions

EA conceived the idea for this study. EA and CØ performed the first analysis together and contributed to the interpretation of the results. EA took the lead in the second analysis with support and input from CØ. EA wrote the manuscript with feedback from CØ. All authors contributed to the article and approved the submitted version.

## Funding

This work was funded by grants from the DAM Foundation through The Norwegian Council for Mental Health, The Norwegian Directorate of Health, and Norwegian Centre for Violence and Traumatic Stress Studies.

## Conflict of interest

The authors declare that the research was conducted in the absence of any commercial or financial relationships that could be construed as a potential conflict of interest.

## Publisher’s note

All claims expressed in this article are solely those of the authors and do not necessarily represent those of their affiliated organizations, or those of the publisher, the editors and the reviewers. Any product that may be evaluated in this article, or claim that may be made by its manufacturer, is not guaranteed or endorsed by the publisher.

## References

[ref1] Andersson E. S. Skar A.-M. S. Jensen T. K. (2021). Unaccompanied refugee minors and resettlement: turning points towards integration. Eur. J. Soc. Psychol. 51, 1–13. doi: 10.1002/ejsp.2761

[ref2] Andreasson U. (2017). Tillit – det nordiska guldet. Denmark: Nordiska Ministerrådet.

[ref3] Andreouli E. (2013). Identity and acculturation: the case of naturalised citizens in Britain. Cult. Psychol. 19, 165–183. doi: 10.1177/1354067X13478984

[ref4] Ball E. Steffens M. C. Niedlich C. (2022). Racism in Europe: characteristics and Intersections With Other Social Categories. Front. Psychol. 13:789661. doi: 10.3389/fpsyg.2022.789661, PMID: 35401357 PMC8988036

[ref5] Bean T. Derluyn I. Eurelings-Bontekoe E. Broekaert E. Spinhoven P. (2007). Comparing psychological distress, traumatic stress reactions, and experiences of unaccompanied refugee minors with experiences of adolescents accompanied by parents. J. Nerv. Ment. Dis. 195, 288–297. doi: 10.1097/01.nmd.0000243751.49499.93, PMID: 17435478

[ref6] Belford N. (2017). International students from Melbourne describing their cross-cultural transitions experiences: culture shock, social interaction, and friendship development. J. Int. Stud. 7, 499–521. doi: 10.32674/jis.v7i3.206

[ref7] Berry J. W. (1997). Immigration, acculturation, and adaptation. Appl. Psychol. Int. Rev. 46, 5–34.

[ref8] Berry J. W. Phinney J. S. Sam D. L. Vedder P. (2006). Immigrant youth: acculturation, identity, and adaptation. Appl. Psychol. Int. Rev. 55, 303–332. doi: 10.1111/j.1464-0597.2006.00256.x, PMID: 37060571

[ref9] Bhatia S. Ram A. (2001). Rethinking ‘acculturation’ in relation to diasporic cultures and postcolonial identities. Hum. Dev. 44, 1–18. doi: 10.1159/000057036

[ref10] Bhatia S. Ram A. (2009). Theorizing identity in transnational and diaspora cultures: a critical approach to acculturation. Int. J. Intercult. Relat. 33, 140–149. doi: 10.1016/j.ijintrel.2008.12.009

[ref11] Braun V. Clarke V. (2006). Using thematic analysis in psychology. Qual. Res. Psychol. 3, 77–101. doi: 10.1191/1478088706qp063oa, PMID: 37067850

[ref12] Braun V. Clarke V. (2021). Can I use TA? Should I use TA? Should I not use TA? Comparing reflexive thematic analysis and other pattern-based qualitative analytic approaches. Couns. Psychother. Res. 21, 37–47. doi: 10.1002/capr.12360, PMID: 37067069

[ref13] Brook M. I. Ottemöller F. G. (2020). A new life in Norway: the adaptation experiences of unaccompanied refugee minor girls. Child Youth Serv. Rev. 117:105287. doi: 10.1016/j.childyouth.2020.105287

[ref14] Cengiz P.-M. Eklund Karlsson L. (2021). Portrayal of immigrants in danish media—a qualitative content analysis. Societies 11:45. doi: 10.3390/soc11020045

[ref15] Cheung B. Y. Chudek M. Heine S. J. (2011). Evidence for a sensitive period for acculturation: younger immigrants report acculturating at a faster rate. Psychol. Sci. 22, 147–152. doi: 10.1177/0956797610394661, PMID: 21189354

[ref16] Chirkov V. (2009). Critical psychology of acculturation: what do we study and how do we study it, when we investigate acculturation? Int. J. Intercult. Relat. 33, 94–105. doi: 10.1016/j.ijintrel.2008.12.004, PMID: 25545573

[ref17] Derluyn I. Lippens V. L. Verachtert T. Bruggeman W. Broekaert E. (2010). Minors travelling alone: a risk group for human trafficking? Int. Migr. 48, 164–185. doi: 10.1111/j.1468-2435.2009.00548.x, PMID: 20645474

[ref18] Doksheim M. (2011). Den nordiske modellen [Online]. www.civita.no: Civita. Available at: https://civita.no/notat/nr-7-2011-den-nordiske-modellen/ (Accessed January 7, 2021).

[ref19] Doksheim M. (2017). Den nordiske modellen [Online]. www.civita.no: Civita. Available at: https://civita.no/politisk-ordbok/hva-er-den-nordiske-modellen/ (Accessed January 7, 2021).

[ref20] Eberl J.-M. Meltzer C. E. Heidenreich T. Herrero B. Theorin N. Lind F.. (2018). The European media discourse on immigration and its effects: a literature review. Ann. Int. Commun. Assoc. 42, 207–223. doi: 10.1080/23808985.2018.1497452

[ref21] EL-Awad U. Fathi A. Vasileva M. Petermann F. Reinelt T. (2021). Acculturation orientations and mental health when facing post-migration stress: differences between unaccompanied and accompanied male Middle Eastern refugee adolescents, first- and second-generation immigrant and native peers in Germany. Int. J. Intercult. Relat. 82, 232–246. doi: 10.1016/j.ijintrel.2021.04.002

[ref22] Esses V. M. Medianu S. Lawson A. S. (2013). Uncertainty, threat, and the role of the media in promoting the dehumanization of immigrants and refugees. J. Soc. Issues 69, 518–536. doi: 10.1111/josi.12027

[ref23] Fedi A. Mannarini T. Brodsky A. Rochira A. Buckingham S. Emery L.. (2019). Acculturation in the discourse of immigrants and receiving community members: results from a cross-national qualitative study. Am. J. Orthopsychiatry 89, 1–15. doi: 10.1037/ort0000325, PMID: 29792478

[ref24] Gamsakhurdia V. (2018). Adaptation in a dialogical perspective - From acculturation to proculturation. Cult. Psychol. 24, 545–559. doi: 10.1177/1354067X18791977

[ref25] Gamsakhurdia V. (2019). Proculturation: self-reconstruction by making fusion cocktails of alien and familiar meanings. Cult. Psychol. 25, 161–177. doi: 10.1177/1354067X19829020

[ref26] Hellevik T. (2005). Ungdom, etablering og ulike velferdsregimer. Tidskrift for Ungdomsforskning 5, 89–110.

[ref27] Hill C. E. Knox S. ThompSON B. J. Williams E. N. Hess S. A. Ladany N. (2005). Consensual qualitative research: an update. J. Couns. Psychol. 52, 196–205. doi: 10.1037/0022-0167.52.2.196, PMID: 36997744

[ref28] Hvinden B. (2009). Den nordiske velferdsmodellen: likhet, trygghet - og marginalisering? Sosiologi i dag 39, 11–36.

[ref29] IMDI (2022). Ny integreringspakke vedtatt [Online]. Available at: https://www.imdi.no/om-imdi/aktuelt-na/ny-integreringspakke-vedtatt--videreforing-av-utvidet-introduksjonsprogram-og-utvidet-norskopplaring/?utm_source=IMDI+-+Nyhetsbrev&utm_campaign=49e53e5557-m_COPY_01&utm_medium=email&utm_term=0_d03f2a9490-49e53e5557-153943253 (accessed July 8, 2022).

[ref30] Jensen T. K. Fjermestad K. W. Granly L. Wilhelmsen N. H. (2015). Stressful life experiences and mental health problems among unaccompanied asylum-seeking children. Clin. Child Psychol. Psychiatry 20, 106–116. doi: 10.1177/1359104513499356, PMID: 23982990

[ref31] Jensen T. K. Skar A.-M. S. Andersson E. S. Birkeland M. (2019). Long-term mental health in unaccompanied refugee minors: pre- and post-flight predictors. Eur. Child Adolesc. Psychiatry 28, 1671–1682. doi: 10.1007/s00787-019-01340-6, PMID: 31004294

[ref32] Jore T. Oppedal B. Biele G. (2020). Social anxiety among unaccompanied minor refugees in Norway. The association with pre-migration trauma and post-migration acculturation related factors. J. Psychosom. Res. 136:110175. doi: 10.1016/j.jpsychores.2020.110175, PMID: 32652372

[ref33] Keles S. Friborg O. Idsøe T. Sirin S. Oppedal B. (2016). Depression among unaccompanied minor refugees: the relative contribution of general and acculturation-specific daily hassles. Ethn. Health 21, 300–317. doi: 10.1080/13557858.2015.1065310 26208789

[ref34] Luster T. Qin D. Bates L. Rana M. Jung A. L. (2010). Successful adaptation among Sudanese unaccompanied minors: perspectives of youth and foster parents. Childhood 17, 197–211. doi: 10.1177/0907568210365664

[ref35] Märtsin M. Mahmoud H. W. (2012). “Never at-Home?: migrants between Societies” in The Oxford Handbook of Culture and Psychology. ed. Valsiner J. (New York: Oxford University Press)

[ref36] Müller L. R. F. Büter K. P. Rosner R. Unterhitzenberger J. (2019). Mental health and associated stress factors in accompanied and unaccompanied refugee minors resettled in Germany: a cross-sectional study. Child Adolesc. Psychiatry Ment. Health 13:8. doi: 10.1186/s13034-019-0268-1, PMID: 30719070 PMC6352340

[ref37] Nordic Co-operation. (n.d.). Nordic co-operation on integration [Online]. Nordisk ministerråd. Available at: https://www.norden.org/en/information/nordic-co-operation-integration (Accessed July 8, 2022).

[ref38] Nordisk Ministerråd (2020). Norden som verdens mest bærekraftige og integrerte region - Handlingsplan 2021–2024 Danmark: Nordisk Ministerråd.

[ref39] Norredam M. Nellums L. Nielsen R. S. Byberg S. Petersen J. H. (2018). Incidence of psychiatric disorders among accompanied and unaccompanied asylum-seeking children in Denmark: a nation-wide register-based cohort study. Eur. Child Adolesc. Psychiatry 27, 439–446. doi: 10.1007/s00787-018-1122-3 29488029

[ref40] Oppedal B. Idsoe T. (2015). The role of social support in the acculturation and mental health of unaccompanied minor asylum seekers. Scand. J. Psychol. 56, 203–211. doi: 10.1111/sjop.12194, PMID: 25614276

[ref41] Pfeiffer E. Behrendt M. Adeyinka S. Devlieger I. Rota M. Uzureau O.. (2022). Traumatic events, daily stressors and posttraumatic stress in unaccompanied young refugees during their flight: a longitudinal cross-country study, 26. Child Adolesc. Psychiatry Ment. Health 16. doi: 10.1186/s13034-022-00461-2, PMID: PMC8974188 35361239

[ref42] Pumariega A. J. Rothe E. (2010). Leaving no children or families outside: the challenges of immigration. Am. J. Orthopsychiatry 80, 505–515. doi: 10.1111/j.1939-0025.2010.01053.x, PMID: 20950291

[ref43] Ryan D. Dooley B. Benson C. (2008). Theoretical perspectives on post-migration adaptation and psychological well-being among refugees: towards a resource-based model. J. Refug. Stud. 21, 1–18. doi: 10.1093/jrs/fem047

[ref44] Skivenes M. (2020). Ta barns rettigheter på alvor [Online]. Forskning. Available at: https://blogg.forskning.no/blogg-stat-og-individ/ta-barns-rettigheter-pa-alvor/1650716.

[ref45] Smith J. A. Flowers P. Larkin M. (2009). Interpretative phenomenological analysis. London, SAGE Publications.

[ref46] Ungar M. (2008). Resilience across Cultures. Br. J. Soc. Work 38, 218–235. doi: 10.1093/bjsw/bcl343

[ref47] Ungar M. (2011). The social ecology of resilience: addressing contextual and cultural ambiguity of a nascent construct. Am. J. Orthopsychiatry 81, 1–17. doi: 10.1111/j.1939-0025.2010.01067.x, PMID: 21219271

[ref48] UNICEF. (2022). Child displacement [Online]. Available at: https://data.unicef.org/topic/child-migration-and-displacement/displacement/ (Accessed July 12, 2022).

[ref49] Vervliet M. Lammertyn J. Broekaert E. Derluyn I. (2014). Longitudinal follow-up of the mental health of unaccompanied refugee minors. Eur. Child Adolesc. Psychiatry 23, 337–346. doi: 10.1007/s00787-013-0463-1, PMID: 23979476

[ref50] Ward C. (2008). Thinking outside the Berry boxes: new perspectives on identity, acculturation and intercultural relations. Int. J. Intercult. Relat. 32, 105–114. doi: 10.1016/j.ijintrel.2007.11.002

[ref51] Woodgate R. L. Busolo D. S. (2021). African refugee youth’s experiences of navigating different cultures in canada: a push and pull experience. Int. J. Environ. Res. Public Health 18:2063. doi: 10.3390/ijerph18042063, PMID: 33672518 PMC7923778

